# Exploration of the Microstructure and Rheological Properties of Sodium Alginate-Pectin-Whey Protein Isolate Stabilized Β-Carotene Emulsions: To Improve Stability and Achieve Gastrointestinal Sustained Release

**DOI:** 10.3390/foods10091991

**Published:** 2021-08-25

**Authors:** Haoxin Ye, Tingshuai Chen, Min Huang, Gerui Ren, Qunfang Lei, Wenjun Fang, Hujun Xie

**Affiliations:** 1 School of Food Science and Biotechnology, Zhejiang Gongshang University, Hangzhou 310018, China; haosyzjsu@163.com (H.Y.); chentingshuai1227@163.com (T.C.); rengerui@zjgsu.edu.cn (G.R.); hujunxie@gmail.com (H.X.); 2 Department of Chemistry, Zhejiang University, Hangzhou 310027, China; qflei@zju.edu.cn (Q.L.); fwjun@zju.edu.cn (W.F.)

**Keywords:** β-carotene, polysaccharide, protein, encapsulation, emulsion

## Abstract

Sodium alginate (SA)-pectin (PEC)-whey protein isolate (WPI) complexes were used as an emulsifier to prepare β-carotene emulsions, and the encapsulation efficiency for β-carotene was up to 93.08%. The confocal laser scanning microscope (CLSM) and scanning electron microscope (SEM) images showed that the SA-PEC-WPI emulsion had a compact network structure. The SA-PEC-WPI emulsion exhibited shear-thinning behavior and was in a semi-dilute or weak network state. The SA-PEC-WPI stabilized β-carotene emulsion had better thermal, physical and chemical stability. A small amount of β-carotene (19.46 ± 1.33%) was released from SA-PEC-WPI stabilized β-carotene emulsion in simulated gastric digestion, while a large amount of β-carotene (90.33 ± 1.58%) was released in simulated intestinal digestion. Fourier transform infrared (FTIR) experiments indicated that the formation of SA-PEC-WPI stabilized β-carotene emulsion was attributed to the electrostatic and hydrogen bonding interactions between WPI and SA or PEC, and the hydrophobic interactions between β-carotene and WPI. These results can facilitate the design of polysaccharide-protein stabilized emulsions with high encapsulation efficiency and stability for nutraceutical delivery in food and supplement products.

## 1. Introduction

β-Carotene is a very important nutrient and pigment in human health. As a vitamin A source, β-carotene is an effective plant medicine for dry eye [1]. Meanwhile, it can reduce the incidence rate of macular disease [2] and can help prevent oral cancer [3]. However, the dispersion of β-carotene in water is poor. The stability of β-carotene is low and can easily be degraded by photooxidation and thermal oxidation. Moreover, the bioavailability of β-carotene directly from food is very low because its absorption effect in the gastrointestinal tract is not good [4]. Thus, some strategies including emulsion, nanoparticle and liposome have been applied to improve the stability and bioavailability of β-carotene [5,6,7,8,9,10,11].

Emulsion is a stable dispersion system composed of multiple immiscible liquids. The inner phase is dispersed in the continuous outer phase in the form of droplets. Emulsions have been used to encapsulate drugs [12], nutrients [13] and probiotics [14], some of which have cell-specific target effects [15,16]. For β-carotene, the emulsions encapsulating β-carotene have better solubility in water, and their chemical, physical, and storage stabilities can be increased at the same time [17,18]. In this way, they can achieve the purpose of accurate release in the intestines without being inactivated in the stomach [19,20].

Among the β-carotene encapsulating systems, the polysaccharide-protein coating systems have attracted wide attention. Polysaccharides including modified starch [21], pectin [22], and sodium alginate [23] were often used to encapsulate β-carotene, but the bioavailability was not very high. To improve the bioavailability, different protein was added into the polysaccharide system [24]. For example, the WPI-dextran conjugates stabilized β-carotene emulsion effectively improved the physical and chemical stability of β-carotene [25]. The caseinate/alginate complexes were used to encapsulate β-carotene and the bioavailability and stability of β-carotene were improved after encapsulation [26]. The starch-WPI microgel was chosen to encapsulate β-carotene and achieved sustained release of β-carotene in the simulated gastrointestinal tract [24]. Thus, the WPI-polysaccharide systems are effective in protecting β-carotene and achieving its accurate release. However, most research on β-carotene encapsulation found it difficult to achieve the high encapsulation efficiency, high stability, and gastrointestinal sustained release of β-carotene at the same time. How to further realize these goals of remains difficult.

Pectin (PEC) is a type of soluble dietary polysaccharide which exists in higher plant cell walls. PEC is relatively stable in acidic solutions. Its molecule consists of three structural regions, including homogalacturonan (HG), rhamngalacturonan I (RG-I), and rhamngalacturonan II (RG-II). HG is widely known for calcium-mediated gel formation, and pectin can be used as thickener, stabilizer, and gel in food [27]. According to the degree of methoxylation (DM), pectin can be divided into high methoxy pectin (DM > 50%) and low methoxy pectin (DM < 50%). The high methoxy pectin can form a gel under acidic conditions, while low methoxy pectin requires divalent metal ions to produce a gel [28].

Sodium alginate (SA) is a kind of natural edible polysaccharide and is composed of β-D-mannuronic acid (M) and α-L-guluronic acid (G). The carboxyl group of its molecular chain can form a gel by interacting with Ca^2+^ ions [29]. The gel mechanism is the same as that of the low methoxy pectin [30] because the G segment of sodium alginate is almost the same as the HG region of pectin. Sodium alginate can be used to encapsulate small molecules and has many advantages. Previous works showed that sodium alginate encapsulated curcumin has a slow-release effect [31,32]. The Pickering emulsions stabilized by gliadin-sodium alginate coacervate particles have high oil encapsulation efficiency [33].

In order to improve the stability of β-carotene and achieve its gastrointestinal sustained release, the SA-PEC-WPI complexes were used as an emulsifier to prepare β-carotene emulsions. The microstructure, rheological properties, thermal stability, physical stability, chemical stability, and the gastrointestinal release behavior were investigated. The possible interactions for the formation of emulsions were also explored. These results can help us facilitate the design of polysaccharide-protein stabilized emulsions with high encapsulation efficiency and stability, and achieve nutraceutical delivery in food and supplement products.

## 2. Materials and Methods

### 2.1. Materials

High methoxy pectin (galacturonic acid > 74%, Mw = 736889), sodium alginate (AR), β-carotene (>96%, Mw = 536.87), n-hexane (>99%), NaOH (AR, 96%), HCl (≥98%), citrate (AR, ≥98%), sodium citrate monobasic (99%), trypsin (≥250 U/mg), and pepsin (≥2500 U/mg) were derived from Aladdin Co., Ltd. (Shanghai, China). Whey protein isolate (80%) was bought from Shanghai Yuanye Bio-Technology Co., Ltd. (Shanghai, China). Anhydrous ethanol (≥99.7%) was purchased from Xilong Scientific Co., Ltd. (Shantou, China). The water used in the experiment was deionized.

### 2.2. Preparation of SA-PEC-WPI Stabilized β-Carotene Emulsions

The SA, PEC and SA-PEC aqueous solution was prepared. The different ratios of SA and PEC solid powder (1:0, 1:3, 1:1, 3:1, 0:1, *w*/*w*) were dissolved in the citric acid-sodium citrate buffer (0.1 M, pH = 6.0) to reach the final polysaccharide concentration of 1.0 wt%. This acidic condition was conducive to the formation of a gel network of high methoxyl pectin. The solution was stirred at 280 rpm for 4 h at 25 °C. Then, the WPI was dissolved in the citric acid-sodium citrate buffer (0.1 M, pH = 6.0) to make the WPI solution at a protein concentration of 2.0 wt%. The solution was stirred at 440 rpm for 4 h at room temperature. Next, the β-carotene oil solutions were prepared by dissolving different quantities of β-carotene into the corn oil at 60 °C (the final β-carotene concentration was 1, 2, 3, 4, 5, and 6 mg/L, respectively).

The β-carotene oil solution was added into WPI aqueous solution at an oil-to-water ratio of 1:19 (*w*/*w*). The mixture was sheared by a high-speed digital shearer (FJ200-S, Qiwei Instrument Co., Ltd., Hangzhou, China) for 8 min and then homogenized via a high-pressure homogenizer (JJ-30L/60, Shengtong Instrument Co., Ltd., Langfang, China) 3 times at 40 MPa. The WPI-β-carotene emulsion was added to the SA, PEC and SA-PEC aqueous solution at a ratio of 1:4 (*w*/*w*), respectively, followed by homogenization at 40 MPa twice. The SA-WPI, PEC-WPI and SA-PEC-WPI stabilized β-carotene emulsions were obtained. The pH values of systems (2.8, 3.2, 3.6, 4.0, 4.4, 4.8, 5.2, 5.6, 6.0) were adjusted with NaOH and HCl solution.

### 2.3. Measurement of Zeta Potential and Particle Size

The zeta potential and particle size were measured by a Nano-ZS laser particle size analyzer (Malvern, UK). All freshly prepared samples were diluted 100 times in citric acid-sodium citrate buffer to avoid the multiple scattering effect [24]. All experiments were performed at 25 °C and a refractive index of 1.45 [34]. Each test was scanned 12 times and repeated 3 times.

### 2.4. Encapsulation Efficiency and Loading Capacity

The encapsulation efficiency (EE) and loading capacity (LC) of SA-PEC-WPI stabilized β-carotene emulsions at different β-carotene concentrations (10, 20, 30, 40, 50, and 60 mg/L) were studied, respectively. The EE and LC were calculated by Equations (1) and (2) [35].

(1) $$\mathrm{EE}\;\left(\%\right)=\frac{\mathrm{Total}\left(\beta -\mathrm{Carotene}\right)-\mathrm{Free}\left(\beta -\mathrm{carotene}\right)}{\mathrm{Total}\left(\beta -\mathrm{Carotene}\right)}\times 100$$

(2) $$\mathrm{LC}\;\left(‰\right)=\frac{\mathrm{Total}\left(\beta -\mathrm{Carotene}\right)-\mathrm{Free}\left(\beta -\mathrm{carotene}\right)}{\mathrm{Weight}\left(\mathrm{SA}-\mathrm{PEC}-\mathrm{WPI}\;\mathrm{complex}\right)}\times 1000$$

The free β-carotene content was measured using the following method. The n-hexane (3 mL) was added to 1 mL emulsion and then the mixtures were vortexed for 3 min. The mixtures were centrifuged and the supernatant (hexane solution with β-carotene) was taken to measure the absorbance at 450 nm by an ultraviolet spectrophotometer (UV-2600, Shimadzu Instrument Co., Ltd., Suzhou, China). Free β-carotene concentration was determined based on the standard curve shown in Figure S1 (see Supplementary Materials).

### 2.5. Microstructure Analysis

The microstructure of emulsions was observed by CLSM (Leica TCS SP2, Wetzlar, Germany). The Nile blue A solution (0.1 mL, 1 mg/mL) and the Nile red solution (0.1 mL, 1 mg/mL) were added to the sample (2 mL) to dye the protein phase and the oil phase, respectively. The samples were excited with fluorescence at 488 nm and 633 nm to obtain the signals of Nile red and Nile blue A dyes, respectively.

The morphology of emulsions was analyzed by SEM (ZEISS GeminiSEM 300, Jena, Germany) with samples mounted on aluminum stubs and sputtered with gold.

### 2.6. Rheological Behavior of Emulsions

According to the previous method [36], the rheological measurement was performed at 25 °C by a MARS III rheometer (Thermo Fisher Scientific, Waltham, MA, USA) equipped with a Peltier stage enabling temperature control within ±0.5 °C. The cone has a diameter of 60 mm and a gap of 0.100 mm. For each measurement, 3.0 mL emulsion was carefully deposited on the rheometer platform. A thin layer of silicone oil covered the exposed surface of samples to prevent evaporation during measurement. All samples were allowed to rest for 5 min after loading to allow temperature equilibration and induced stress in order to relax.

Steady-state flow measurements were carried out in the range of 5–100 s^−1^, and the rheological parameters (shear rate, apparent viscosity) were obtained. The power-law model was used to fit experimental flow curves, represented by Equation (3).

(3) $$\eta =K{\dot{\gamma }}^{n-1}$$

where *η* is the apparent viscosity (Pa s), $\dot{\gamma }$ is the shear rate (s^−1^), *K* is the consistency index (Pa s^n^), and *n* is the power law index.

Oscillatory frequency sweep experiments were carried out in the angular frequency range of 1–100 rad/s at 25 °C. The linear visco-elastic region was determined to be at 1% strain by strain sweep tests in the strain range of 0.01–100%. The elastic modulus (*G*′) and loss modulus (*G*″) were obtained from the frequency sweep profiles.

### 2.7. Stability Analysis

#### 2.7.1. Thermal Stability of Emulsions

The thermal stability of SA-WPI, SA-PEC-WPI, and PEC-WPI stabilized β-carotene emulsions were analyzed under three different conditions based on a previous method [37] with some modification. First, samples were put into a test tube and then heated in an electric constant temperature water bath (DK-8D, Jinghong Instrument Co., Ltd., Shanghai, China). The conditions were set as 37 °C for 1 h, 60 °C for 30 min, and 90 °C for 10 min, respectively. Samples were cooled to room temperature after heat treatment, and then their particle size and zeta potential were measured.

#### 2.7.2. Physical Stability

A Turbiscan Lab (Formulaction, Toulouse, France) was used to evaluate the physical stability of emulsions. Samples were transferred to a glass cylindrical test tube for analysis. The pulsed near-infrared light wavelength (880 nm) emitted by the sample test tube itself was scanned. Two synchronous optical detectors collected the backscattered light and the transmitted light, respectively. Samples were scanned in the test tube every 15 min at 55 °C for 5 h. The change in BS (ΔBS) in unit time was used to show the oil droplet migration and the creaming in emulsions.

In order to quantify the destabilization processes in emulsions, Turbiscan Stability Index (TSI) was used. This index is a statistical factor, and its value is calculated as the sum of all physical destabilization processes in the measuring tube, and is given below:

(4) $$TSI=\underset{j}{\sum }\left|scan_{ref}\left(h_{j}\right)-scan_{i}\left(h_{j}\right)\right|$$

where *scan_ref_* is the initial backscattering value and *scan_i_* is the backscattering value at a given time, *h_j_* is a given height in the measuring cell and *TSI* is the sum of all the scanning differences from the bottom to the top of the vial [38].

#### 2.7.3. Chemical Stability

The chemical stability of SA-WPI, SA-PEC-WPI, and PEC-WPI stabilized β-carotene emulsions was studied by preserving at 4 °C for 30 d, 25 °C for 30 d, and 60 °C for 5 d, respectively. The retained β-carotene concentration determined the degree of the degradation of β-carotene during the entire storage period. After adding ethanol (2 mL) and n-hexane (3 mL) to each sample (1 mL), the mixtures were vortexed and then stood until the complete phase separation occurred. The upper yellow supernatant (n-hexane phase) was transferred to a 10 mL centrifuge tube. Extraction was repeated twice with 3 mL of n-hexane until the ethanol phase was completely colorless. The mixtures were centrifuged at 4000× *g* for 5 min to separate any insoluble matter. The analysis of each sample was repeated three times, and the absorbance was measured at 450 nm using an ultraviolet spectrophotometer. The retention rate of β-carotene was expressed by Equation (5).

(5) $$\mathrm{Retention}\;\mathrm{rate}\;\left(\%\right)=\frac{C_{t}}{C_{0}}\times 100$$

where *C_t_* is the concentration of β-carotene after a storage period (mg/L), and *C*_0_ is the initial concentration of β-carotene (mg/L).

### 2.8. In Vitro Release Study

The in vitro simulated digestion model was established as described in a previous study [39]. The NaCl (0.2 g) and pepsin (0.32 g) powder were first added to a small amount of deionized water. Then 0.7 mL HCl was added into the solution. The total volume was diluted to 100 mL with deionized water to obtain the simulated gastric fluid (SGF). To obtain the simulated intestinal fluid (SIF), 100 mL aqueous solution with concentrations of 15.36 mM NaCl, 0.15 mM CaCl_2_, 5 mg/mL pig bile salt and 8 mg/mL trypsin was prepared and centrifuged (5000× *g*, 4 °C, 10 min). The SGF, SIF, and SA-PEC-WPI stabilized β-carotene emulsion were preheated in a water bath at 37 °C. Then the emulsion was added to SGF in the volume ratio of 1:1, and the pH value of mixture was rapidly adjusted to 2. The mixture was placed in a gas bath thermostatic oscillator (THZ-C, Jingqi Instrument Co., Ltd., Shanghai, China) (37 °C, 100 rpm) to stimulate the peristalsis of the stomach. After digestion by SGF for 60 min, the chyme from the gastric phase was mixed with preheated SIF in a volume ratio of 1:1. The pH value of this system was rapidly adjusted to 7, and samples were put into the air bath thermostatic oscillator (37 °C, 270 rpm, 120 min). According to the pre-set time interval, 1 mL of sample was collected each time and transferred into a centrifuge tube. The free β-carotene concentration of emulsion was measured. The SA-PEC-WPI emulsion without β-carotene was used as a blank control sample. The cumulative release rate of β-carotene was calculated by Equation (6).

(6) $$\mathrm{Cumulative}\;\mathrm{release}\;\mathrm{rate}\;\left(\%\right)=\frac{C\times V}{M}\times 100$$

where *C* represents the concentration of β-carotene released in the mixed solution (mg/L), *V* represents the volume of the solution (mL), and *M* represents the amount of β-carotene contained in the emulsion (mg).

### 2.9. FTIR Spectra

All emulsions were freeze-dried to get solid powders. The mass ratio of the sample was controlled: KBr as 1:100, and the ground mixed powders were pressed into a uniform transparent flake by a tableting press. Samples were scanned 16 times in the wavelength range of 4000–400 cm^−1^ at room temperature using a Fourier transform spectrophotometer (Nicolet iS5, Thermo Fisher, New York, NY, USA). Each sample was repeated three times.

### 2.10. Statistical Analysis

All experiments were carried out three times. The results were expressed as mean ± standard deviation (SD). One-way ANOVA was carried out on the data using SPSS 18.0 software (IBM Corp., Armonk, NY, USA). The significance level was less than 5%.

## 3. Results and Discussion

### 3.1. Emulsion Preparation

**Figure 1:**
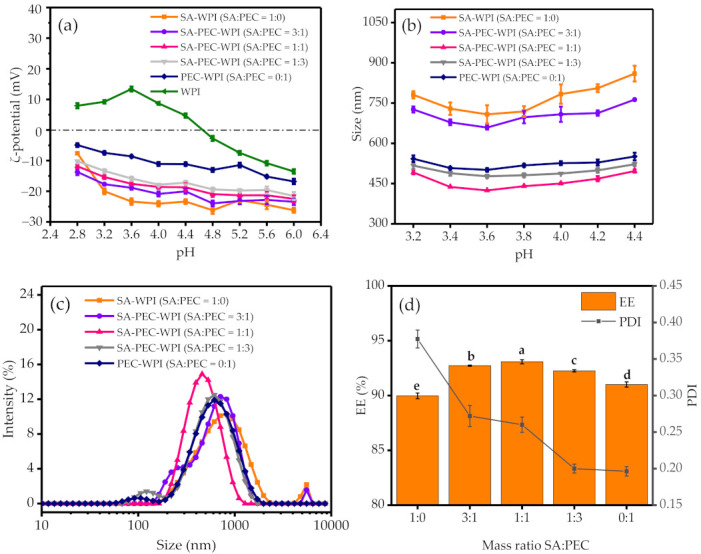
The zeta potential (**a**) and particle size (**b**) of emulsions at different SA:PEC mass ratios and pH values. The particle size distribution (**c**) of different emulsions at pH 3.6. The EE and PDI (**d**) of SA-PEC-WPI emulsions with β-carotene concentration of 40 mg/L at pH 3.6. The different letters (a–e) within figure indicate significant differences (*p* ≤ 0.05). EE: encapsulation efficiency; PDI: polymer dispersity index; SA: sodium alginate; PEC: pectin; WPI: whey protein isolate.

According to Figure 1a, along with the decrease of pH value, the absolute values of zeta potential of SA-WPI, SA-PEC-WPI and PEC-WPI stabilized emulsions decreased, while the absolute values of zeta potential of WPI stabilized emulsion increased first and then decreased. When the pH value was less than 4.4, the zeta potential of WPI stabilized emulsion was positive. When the pH value was more than 3.2, the SA-WPI, SA-PEC-WPI and PEC-WPI stabilized emulsions had a high absolute value of zeta potential. Therefore, the pH values from 3.2 to 4.4 were chosen to study the polysaccharide-protein stabilized emulsions.

Figure 1b shows the particle size changes of all emulsions at a pH range of 3.2–4.4. Along with the increase of pH value, the particle size of emulsions first decreased and then increased. The decrease of particle size was ascribed to the decreased number of protonated carboxyl groups in SA and PEC when the pH value increased, leading to the difficulty of polysaccharide aggregation. The increase of particle size was due to the easy aggregation and precipitation of proteins when the pH value became nearer to the isoelectric point of WPI (4.6). All emulsions had relatively smaller particle sizes at pH 3.6. Thus, pH 3.6 was chosen as the critical pH value for further analysis.

Figure 1c shows the particle size distribution of SA-PEC-WPI emulsions at different SA:PEC mass ratios at pH 3.6. When the mass ratio of SA:PEC was 1:1, the emulsion showed a good unimodal distribution and had a large peak intensity with a peak center at about 424 nm. Furthermore, it can be seen from Figure 1d that the emulsion had the highest EE when the mass ratio of SA:PEC was 1:1. Obviously, an appropriate mass ratio of SA:PEC can improve the encapsulation efficiency of emulsion. Initially, at a relatively higher SA:PEC mass ratio, the emulsion distribution was homogeneous (PDI becomes smaller) and the EE increased to its highest value at ratio of 1:1. With further reduction of the mass ratio of SA:PEC, the EE was decreased because of the formation of a poor gel barrier [40]. Thus, the optimized condition for preparing emulsion was obtained at pH 3.6 with a SA:PEC mass ratio of 1:1.

### 3.2. Encapsulation Efficiency and Loading Capacity

**Figure 2:**
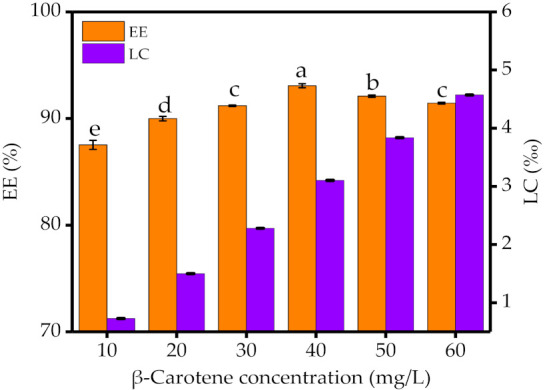
The EE and LC of SA-PEC-WPI stabilized emulsions at different β-carotene concentrations. EE: encapsulation efficiency; LC: loading capacity. The different letters within figure indicate significant differences (*p* ≤ 0.05).

Figure 2 shows the EE and LC of SA-PEC-WPI stabilized β-carotene emulsions at different concentrations of β-carotene. With the increase of β-carotene concentration, the EE of all emulsions increased first and then decreased, while the LC kept increasing. When the concentration of β-carotene reached 40 mg/L, the emulsion had the highest EE of 93.08% and a high LC of 3.10‰. The results indicated that it was difficult to dissolve β-carotene in the citric acid-sodium citrate buffer, while the SA-PEC-WPI stabilized β-carotene emulsion was well dispersed in the buffer, indicating that the interaction between SA-PEC-WPI and β-carotene can promote the dissolution of β-carotene in the buffer to increase the EE of β-carotene.

### 3.3. Microstructure Analysis

**Figure 3:**
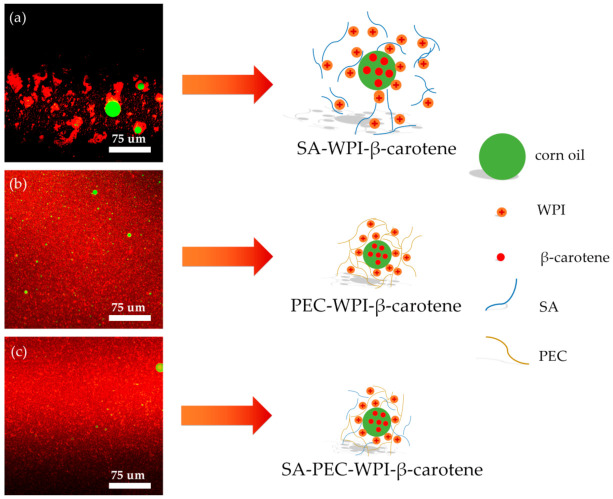
The CLSM images of SA-WPI (**a**), PEC-WPI (**b**), and SA-PEC-WPI (**c**) stabilized β-carotene emulsions. CLSM: confocal laser scanning microscope; SA: sodium alginate; PEC: pectin; WPI: whey protein isolate.

The results of CLSM are shown in Figure 3. The oil phase was dyed green and the protein phase was dyed red. The CLSM images showed an effective combination of corn oil and WPI, indicating that WPI has a good emulsifying ability to stabilize the oil–water interface of emulsions [41]. The apparent aggregation of droplets was found in the SA-WPI emulsion, which can be explained by a bridging flocculation mechanism [42]. Compared to the SA-WPI emulsion, the particle size of PEC-WPI and SA-PEC-WPI emulsions was much smaller and the distribution of droplets was more uniform, which was consistent with the results from the particle size measurement.

**Figure 4:**
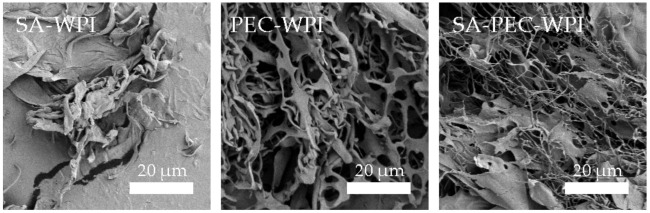
The SEM images of SA-WPI, PEC-WPI, and SA-PEC-WPI stabilized β-carotene emulsions. SEM: scanning electron microscope; SA: sodium alginate; PEC: pectin; WPI: whey protein isolate.

Figure 4 shows the SEM images of SA-WPI, PEC-WPI and SA-PEC-WPI stabilized β-carotene emulsions. The SA-WPI emulsion had no obvious network structure due to the aggregation of droplets in the system. The PEC-WPI and SA-PEC-WPI emulsions had a prominent network structure, indicating better creaming stability of the emulsions [37]. The network structure of SA-PEC-WPI emulsion had relatively more compact pores, mainly because the addition of SA was conducive to the formation of a compact structure. The combination of SA and PEC can form a compact spherical structure by interacting with Ca^2+^ ions which has been used to encapsulate ciprofloxacin (a hydrophobic antibiotic), achieving the effect of slow release in the gastrointestinal tract [40]. In addition, previous studies have shown that the O/W emulsions in the presence of chitosan with a compact network have high stability [43]. Thus, the network structure in the SA-PEC-WPI emulsion has a potential role in improving the stability of emulsions and achieves gastrointestinal sustained release of β-carotene.

### 3.4. Rheological Properties

#### 3.4.1. Flow Behavior

**Figure 5:**
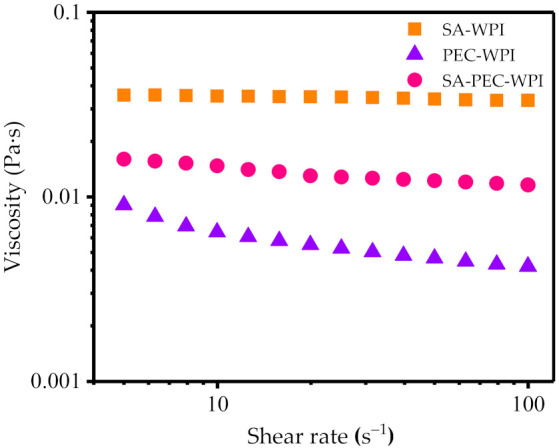
The viscosity of SA-WPI, PEC-WPI, SA-PEC-WPI emulsions. SA: sodium alginate; PEC: pectin; WPI: whey protein isolate.

Viscosity is an essential characteristic of emulsions, related to the rate of creaming and the physical shelf-life of products. Figure 5 shows that the effect of SA-WPI, SA-PEC-WPI, and PEC-WPI complexes on the rheological properties of β-carotene emulsions at 25 °C and pH 3.6. The apparent viscosity of all emulsions decreased slightly with the increase of shear rate in the range of 5–100 s^−1^. As the shear rate increased, the emulsion droplets offered strong resistance to flow, resulting in the inconspicuous change of viscosity. The viscosity of SA-WPI emulsion was higher than that of SA-PEC-WPI and PEC-WPI emulsions (Figure 5). The high viscosity of emulsion could be explained by the occurrence of droplet aggregates and flocculation, caused by the depletion effect [36]. The addition of PEC reduced the viscosity of emulsion, resulting from the reduced aggregation and flocculation of droplets. Thus, the stability of emulsions was increased because of the addition of PEC, consistent with the microstructure observed by CLSM (Figure 3).

The rheological curves were fitted by the power-law model and the relevant rheological parameters were listed in Table 1. Power-law plots showed good fits for the measured data in all tested emulsions (*R*^2^ > 0.9). The *K* value reflects the consistency index which is numerically equal to the viscosity at 1 s^−1^. The increasing viscosity of continuous phase could be found in emulsions with the addition of SA, indicating that SA is conducive to the increase of viscosity. The *n* value is typically an indicator of the fluidity pattern. Generally, the flow is shear-thinning when *n* < 1, Newtonian fluidity when *n* = 1, and shear-thickening when *n* > 1. The *n* values of SA-WPI, PEC-WPI and SA-PEC-WPI emulsions were less than 1, indicating that these emulsions exhibited shear-thinning behavior, especially the PEC-WPI emulsion (*n* = 0.7511). In general, the entangled polymer network in emulsions may be easily disrupted by the increasing shear forces during shearing. However, the network structure formed in SA-PEC-WPI emulsion was more intermolecular resistance to flow at higher shear rates, indicating that this network structure was relatively compact and not easy to deform, which was consistent with the SEM results.

#### 3.4.2. Viscoelastic Properties

**Figure 6:**
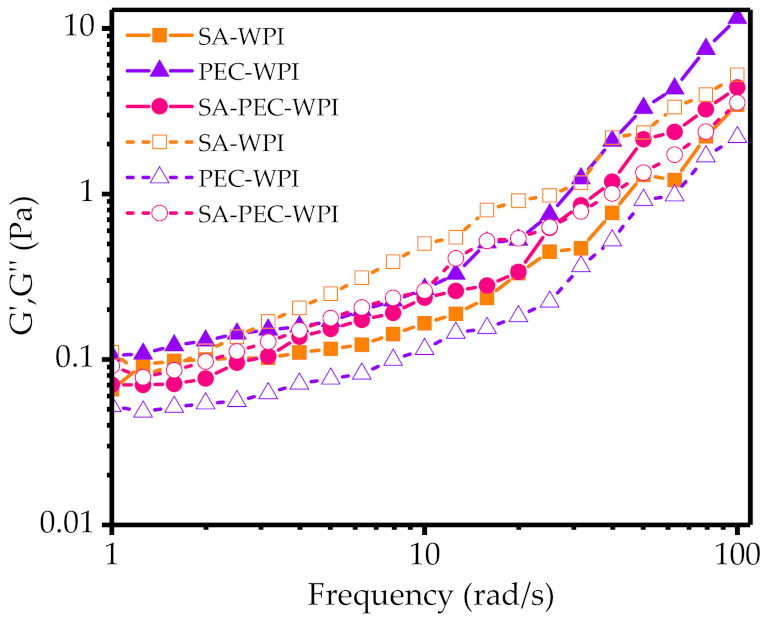
Dynamic frequency sweep of all emulsions. *G*′ in solid line is storage modulus, and *G*″ in dashed line is loss modulus. SA: sodium alginate; PEC: pectin; WPI: whey protein isolate.

The storage modulus (*G*′) and loss modulus (*G*″) of emulsions were measured by oscillatory frequency sweep experiments (Figure 6). In the frequency range of 1–100 rad/s, the *G*′ and *G*″ curves of all emulsions increased with the increase of frequency. The *G*″ value of SA-WPI emulsion was higher than the corresponding *G*′ value, indicating a viscous-dominated behavior. But the PEC-WPI emulsion showed an apparent elastic-dominated behavior (*G*′ is higher than *G*″) in the whole frequency range. In addition, the *G*′ and *G*″ curves of PEC-WPI emulsion were almost parallel to each other, which is a typical characteristic of weak gel [44]. The *G*′ value of PEC-WPI emulsion was higher than that of SA-WPI emulsion, which can be explained by the cohesive/compact structure found at a high concentration of hydrocolloid solution [45]. A crossover of *G*′ and *G*″ values occurred in SA-PEC-WPI emulsion at a high frequency, indicating that an entangled network had been developed [44]. When the oscillating frequency exceeded the crossover frequency, the *G*″ value increased faster than the *G*′ value, suggesting that the SA-PEC-WPI emulsion may be in semi dilute or weak network state [46]. Therefore, the addition of SA resulted in a transition from a predominately elastic to a viscous state, in agreement with results from the steady shear studies.

### 3.5. Emulsion Stability

#### 3.5.1. Heat Stability

In many commercial applications, emulsions need to be heat-treated to prevent microbial contamination. Meanwhile, emulsions will also be affected by heat during the transportation and sales process. Therefore, three heat treatment conditions (37 °C for 1 h, 60 °C for 30 min, and 90 °C for 10 min) were applied to study the effects of heat treatment on the emulsion stability.

**Figure 7:**
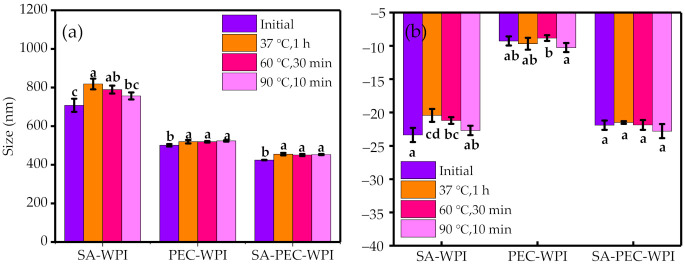
The effect of different thermal treatments on the particle size (**a**) and zeta potential (**b**) of the emulsions. The different letters (a–d) within the figure indicate significant differences (*p* ≤ 0.05).

The three different kinds of heat treatment did not significantly change the particle size and zeta potential of PEC-WPI and SA-PEC-WPI emulsions (Figure 7a,b). These results indicated that these two emulsions had good thermal stability. However, the particle size of SA-WPI emulsion became larger than the control sample after three different kinds of heat treatment. This may be because the creaming or Ostwald ripening easily occurs during the heat treatment process in the SA-WPI emulsion, since its initial particle size was larger than that of PEC-WPI and SA-PEC-WPI emulsions. Previous experiments [47] also revealed that the emulsion with a small particle size has good thermal stability, which was consistent with the results in this study.

#### 3.5.2. Physical Stability

**Figure 8:**
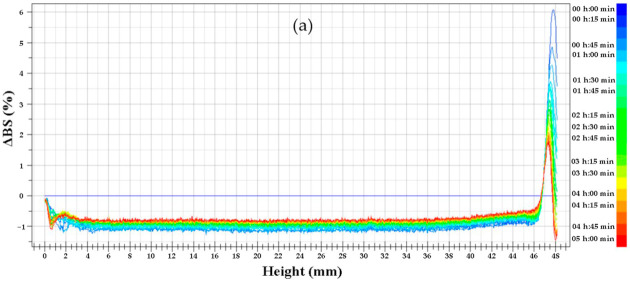

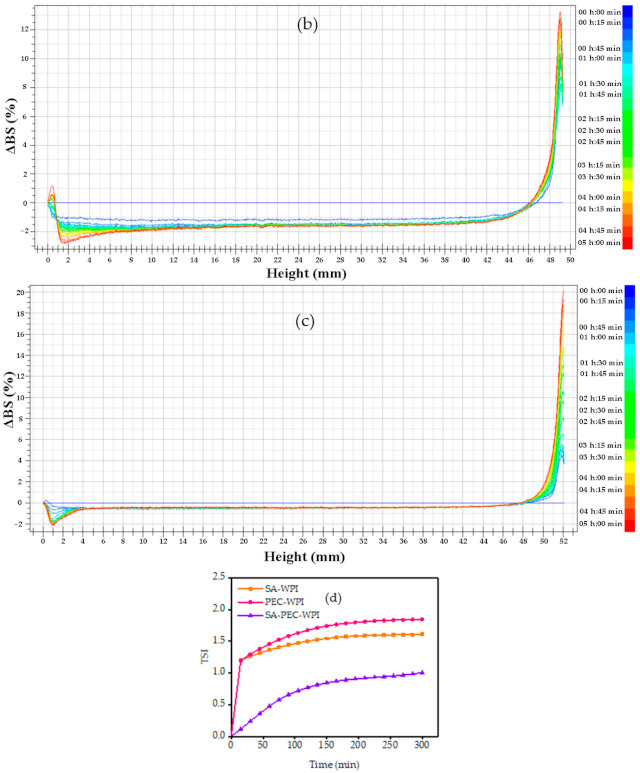
The ΔBS of SA-WPI (**a**), PEC-WPI (**b**) and SA-PEC-WPI (**c**) stabilized β-carotene emulsions, and the changes in TSI values (**d**) during the storage at 55 °C. TSI: Turbiscan stability index; ΔBS: the change in backscattering (BS); SA: sodium alginate; PEC: pectin; WPI: whey protein isolate.

To characterize the physical stability of SA-WPI, PEC-WPI and SA-PEC-WPI stabilized β-carotene emulsions, Turbiscan backscattering data was plotted against sample height over time and shown in Figure 8. The ΔBS level of all emulsions continued to decrease during storage, suggesting that flocculation or coalescence occurred in all samples [48]. Migration of oil droplets causes the decrease of ΔBS in the bottom of the sample due to the decline in oil volume fraction. By contrast, ΔBS in the top of the sample increases owing to the increased oil volume fraction. The ΔBS signal increased at the top of sample tube, suggesting that corn oil migrated from the bottom to the top and a concomitant creaming took place in the lower zone [49]. Compared with the SA-WPI emulsion, the PEC-WPI and SA-PEC-WPI emulsions had network structure, so their creaming was relatively inapparent. The stability against creaming of SA-PEC-WPI emulsion was better than that of PEC-WPI emulsion, which confirmed the conclusion of rheological experiments because the emulsion with high viscosity had better anti-emulsifying strength [50]. The changes in Turbiscan stability index (TSI) were further determined to reflect the destabilization of emulsions through summing up the variations (including creaming, coalescence, and/or flocculation) [38]. The results in Figure 8d show that the SA-PEC-WPI emulsion showed much lower TSI values during the 5 h storage than those of the other two emulsions, suggesting a better physical stability.

#### 3.5.3. Chemical Stability

**Figure 9:**
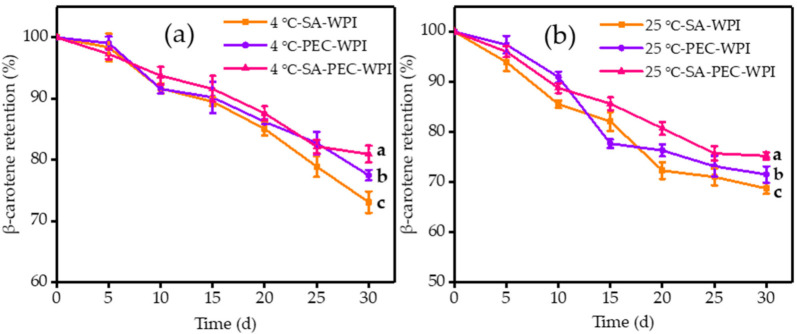

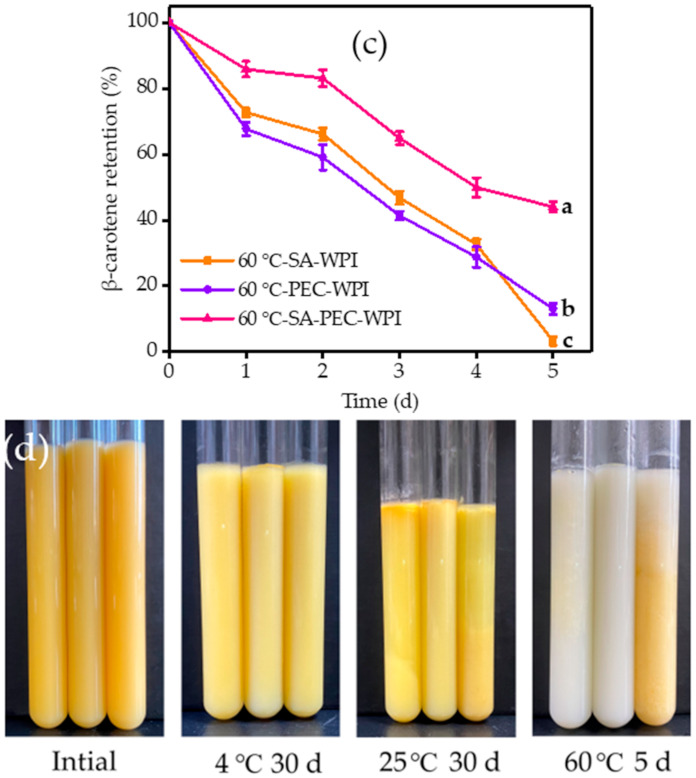
Degradation of β-carotene in the SA-WPI, PEC-WPI, and SA-PEC-WPI emulsions during storage at (**a**) 4 °C for 30 d, (**b**) 25 °C for 30 d, and (**c**) 60 °C for 5 d. (**d**) The change in appearance of the emulsions before and after storage. The different letters (a–c) within the figure indicate significant differences (*p* ≤ 0.05). SA: sodium alginate; PEC: pectin; WPI: whey protein isolate.

β-Carotene is sensitive to free radicals, oxygen, heat and light, which can lead to chemical degradation of polyunsaturated hydrocarbons. Thus, chemical stability is a critical indicator for the SA-WPI, PEC-WPI, and SA-PEC-WPI stabilized β-carotene emulsions. Figure 9 shows the chemical stability of all samples during the respective storage at 4 °C for 30 d, at 25 °C for 30 d, and at 60 °C for 5 d.

The retention rate of β-carotene in the SA-PEC-WPI emulsion was relatively higher than for the other two emulsions at the end of storage (Figure 9a–c). The retention rate of β-carotene (*C*/*C*_0_) in SA-PEC-WPI emulsion fell from an initial value of 100% to 80.97 ± 1.36% after storage at 4 °C for 30 d, to 75.26 ± 0.75% after storage at 25 °C for 30 d, and to 44.02 ± 1.54% after storage at 60 °C for 5 d, respectively. The results showed that the degradation rate of β-carotene increased at higher storage temperature, which was consistent with the previous results [51]. Comparing with the SA-WPI and PEC-WPI emulsions, the high retention rate of SA-PEC-WPI emulsion may be caused by two reasons. On the one hand, the presence of a relatively compact biopolymer layer (SA-PEC-WPI complexes) around the oil droplets provided a steric barrier, which can prevent the diffusion of prooxidants and free radicals from entering the droplets [37]. On the other hand, the SA-PEC-WPI emulsion might have better stability to phase separation, inhibiting the propagating of oxidation from one droplet to another. It can be seen from Figure 9d that no obvious creaming in the three emulsions was observed after storage at 4 °C for 30 d, but all emulsions produced an oil slick phenomenon after storage at 25 °C for 30 d. The SA-WPI and PEC-WPI emulsions almost completely faded after storage at 60 °C for 5 d, while the color of SA-PEC-WPI emulsion remained slightly.

From the upper results, it is obvious that the SA-PEC-WPI stabilized β-carotene emulsion had better thermal stability, physical stability, and chemical stability. Thus, it has potential application in food systems to stabilize β-carotene and achieve commercial values.

### 3.6. In Vitro Sustained Release Behavior

The functional factors in foods are easily digested and metabolized in the gastrointestinal tract because of the presence of many digestive enzymes such as pepsin, trypsin, and salt ions. It is difficult to achieve the goal of targeted release. Therefore, study of the digestive behavior of the SA-PEC-WPI stabilized β-carotene emulsion in the SGF and SIF is of great importance for better understanding its potential application in the food industry.

**Figure 10:**
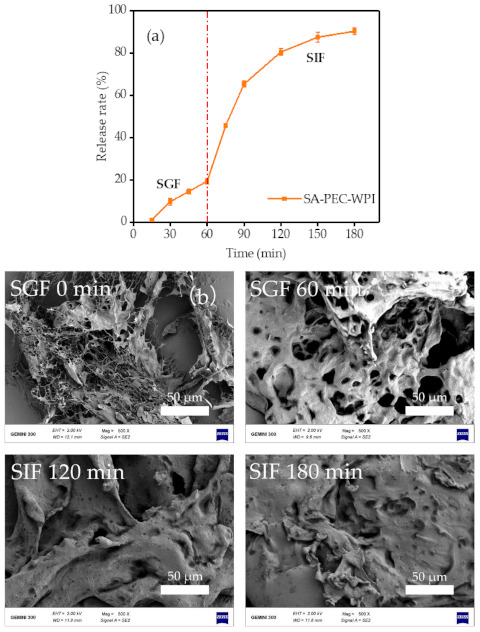
In vitro simulated digestion experiments. (**a**) The release rate of β-carotene from the SA-PEC-WPI stabilized β-carotene emulsion. (**b**) The SEM images of SA-PEC-WPI stabilized β-carotene emulsion at different digestion times. SEM: scanning electron microscope; SGF: simulated grastric fluid; SIF: simulated intestinal fluid; SA: sodium alginate; PEC: pectin; WPI: whey protein isolate.

The cumulative release rate of β-carotene in the emulsion was measured and shown in Figure 10a. After 60 min of SGF digestion, the cumulative release rate of β-carotene was low (only 19.46 ± 1.33%). In the SIF digestion, the cumulative release rate of β-carotene first increased sharply and then became gentle and reached the final cumulative release rate of 90.33 ± 1.58% after 120 min intestinal digestion.

Figure 10b shows the micromorphology change of the SA-PEC-WPI emulsion during in vitro digestion. The network structure of emulsion was retained after 60 min digestion in the SGF, indicating that the polysaccharide chain (SA-PEC complexes) was not well digested in SGF. This result may explain the low accumulative release rate of β-carotene in SGF digestion. On the one hand, the combination of SA-PEC complexes and WPI may protect WPI against pepsin. On the other hand, both pepsin and SA-PEC complexes were negatively charged under the SGF conditions, so the access of pepsin to the underlying positively charged binding points of the WPI layer was restricted because of the electrostatic repulsion [52]. In addition, it can be seen from Figure 10b that the network structure of emulsion after 60 min digestion in SGF swelled when compared with the emulsion before digestion.

However, the SA-PEC-WPI emulsion was easily hydrolyzed in the SIF system. The network structure of this emulsion disappeared after SIF digestion (Figure 10b). Bile salts may have displaced the SA-PEC complexes on the droplet surfaces [53]. In this way, SA-PEC complexes were unable to protect the WPI, leading to its hydrolysis. In addition, the electrostatic repulsion between WPI and SA-PEC complexes resulted in the decrease in binding between WPI and SA-PEC complexes. Thus, the cumulative release rate of β-carotene increased sharply during the SIF digestion.

### 3.7. Emulsion Analysis by FTIR

**Figure 11:**
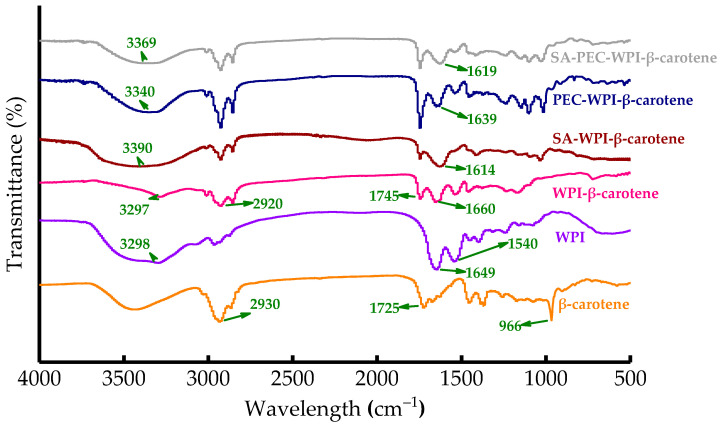
FTIR spectra of β-carotene, WPI and WPI, SA-WPI, PEC-WPI, SA-PEC-WPI stabilized β-carotene emulsions. FTIR: Fourier transform infrared; SA: sodium alginate; PEC: pectin; WPI: whey protein isolate.

In order to explore the molecular mechanism, the FTIR spectra of the freeze-dried WPI, SA-WPI, PEC-WPI, and SA-PEC-WPI stabilized β-carotene complexes were measured and the results are shown in Figure 11. The 966 cm^−1^ characteristic peak of β-carotene depicts the trans conjugated alkene −CH = CH- out-of-plane deformation mode [54], and the 2930 cm^−1^ peak represents the stretching vibration of C-H [55]. WPI is an amphiphilic substance. The characteristic peak of the hydroxyl group in WPI appears at 3298 cm^−1^, and the peaks at 1649 cm^−1^ and 1540 cm^−1^ are the amide I and amide II bands of WPI, respectively [56].

The changes in the C-H stretching frequencies are related to hydrophobic interactions in both the polymer and the low-molecular-weight compounds [57]. The C-H characteristic peak of β-carotene in the WPI stabilized β-carotene emulsion obviously shifted to 2920 cm^−1^, indicating the hydrophobic interaction between C-H in β-carotene and amide I band in WPI. This result might explain the tight combination between WPI and corn oil containing β-carotene observed by CLSM and was consistent with the previous study [58]. Besides, electrostatic interactions can contribute to the amide I band frequency fluctuations [59]. After adding polysaccharides (SA, PEC, or SA-PEC) into the WPI stabilized β-carotene emulsion, the amide I band also changed because of the electrostatic interactions between −COO^-^ in SA or PEC and −NH_3_^+^ in WPI possibly, consistent with zeta potential experiments. Simultaneously, the −OH characteristic peak of WPI was also obviously shifted to the large wavelength, indicating hydrogen bonding interactions between −OH in WPI and the functional groups (−OH, −O-, −COOH) in SA or PEC. Therefore, the hydrophobic interactions, electrostatic interactions, and hydrogen bonding interactions were important for forming SA-PEC-WPI stabilized β-carotene emulsion.

## 4. Conclusions

In this work, the SA-PEC-WPI complexes were used to encapsulate β-carotene. The condition for the preparation of emulsion was optimized by measuring the zeta potential, particle size and EE (pH = 3.6, SA: PEC = 1:1 (*w*/*w*), and the β-carotene concentration = 4 mg/L). The encapsulation efficiency of SA-PEC-WPI emulsion for β-carotene was very high at 93.08%. The SA-PEC-WPI emulsion exhibited shear-thinning behavior and was in a semi-dilute or weak network state. The SA-PEC-WPI emulsion had better physical and chemical stability than SA-WPI and PEC-WPI emulsions with a relatively low TSI value and high retention rate of β-carotene. A small amount of β-carotene was released during simulated gastric digestion, while a large amount of β-carotene was released during simulated intestinal digestion, indicating that the sustained release of β-carotene was achieved. The formation mechanism of SA-PEC-WPI emulsion could be ascribed to the electrostatic and hydrogen bonding interactions between WPI and SA or PEC, and the hydrophobic interactions between β-carotene and WPI. These emulsions can be used in different nutritional foods to achieve sustained release of hydrophobic nutrients and increase the stability of nutrients in food matrices.

## Figures and Tables

**Table 1:** Power-law parameters for the SA-WPI, PEC-WPI, SA-PEC-WPI stabilized β-carotene emulsions.

Samples	*K* (Pa s^n^)	*n*	*R* ^2^
SA-WPI	0.0373 ± 0.0001 ^a^	0.9764 ± 0.0012 ^a^	0.96
PEC-WPI	0.0121 ± 0.0006 ^c^	0.7511 ± 0.0178 ^c^	0.93
SA-PEC-WPI	0.0189 ± 0.0003 ^b^	0.8895 ± 0.0054 ^b^	0.96

Values are means ± standard deviations of triplicates. Superscripts with different letters in same column indicate significant differences (*p* ≤ 0.05). SA: sodium alginate; PEC: pectin; WPI: whey protein isolate.

## Data Availability

Data are available upon request.

## Supplementary Materials

The following are available online at https://www.mdpi.com/article/10.3390/foods10091991/s1, Figure S1: Standard curve of β-carotene.
