# Supplementary figures and images for: Exploration of the Microstructure and Rheological Properties of Sodium Alginate-Pectin-Whey Protein Isolate Stabilized Β-Carotene Emulsions: To Improve Stability and Achieve Gastrointestinal Sustained Release
Source: Foods. 2021 Aug 25;10(9):1991. doi: 10.3390/foods10091991 (PMC8465917; doi:10.3390/foods10091991)

## Supplementary Materials

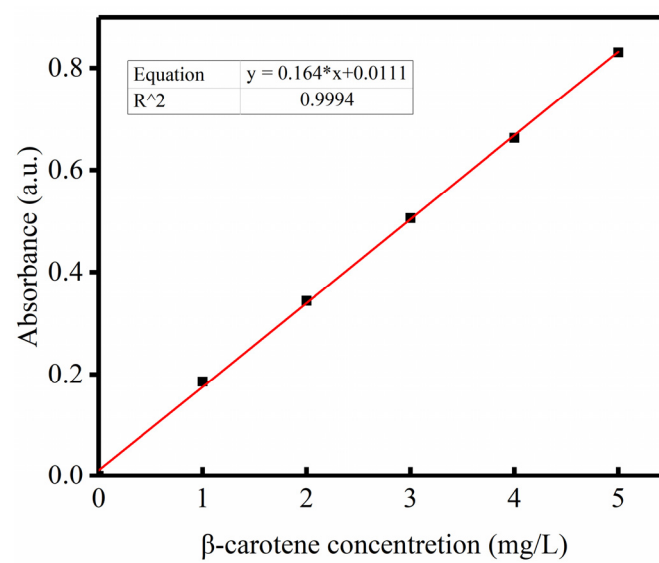

**Figure S1.** Standard curve of β-carotene.

Supplement: Supplementary file 1 [file foods-10-01991-s001.zip › foods-1307557-supplementary.pdf]
